# Hematopoietic Cell Transplantation for MHC Class II Deficiency

**DOI:** 10.3389/fped.2019.00516

**Published:** 2019-12-11

**Authors:** Su Han Lum, Benedicte Neven, Mary A. Slatter, Andrew R. Gennery

**Affiliations:** ^1^Children's Haematopoietic Stem Cell Transplant Unit, Great North Children's Hospital, Newcastle upon Tyne Hospital NHS Foundation Trust, Newcastle upon Tyne, United Kingdom; ^2^Paris Descartes-Sorbonne Paris Cité University, Paris, France; ^3^Pediatric Hematology-Immunology and Rheumatology Unit, Necker-Enfants Malades Hospital, Assistance Publique—Hôpitaux de Paris (APHP), Paris, France; ^4^INSERM U1163 and Imagine Institute, Paris, France; ^5^Institute of Translational and Clinical Research, Newcastle University, Newcastle upon Tyne, United Kingdom

**Keywords:** MHC class II deficiency, children, hematopoietic cell transplantation, transplant strategy, survival

## Abstract

Major histocompatibility complex (MHC) class II deficiency is a rare and fatal primary combined immunodeficiency. It affects both marrow-derived cells and thymic epithelium, leading to impaired antigen presentation by antigen presenting cells and delayed and incomplete maturation of CD4+ lymphocyte populations. Affected children are susceptible to multiple infections by viruses, *Pneumocystis jirovecii*, bacteria and fungi. Immunological assessment usually shows severe CD4+ T-lymphocytopenia, hypogammaglobulinemia, and lack of antigen-specific antibody responses. The diagnosis is confirmed by absence of constitutive and inducible expression of MHC class II molecules on affected cell types which is the immunologic hallmark of the disease. Hematopoietic cell transplantation (HCT) is the only established curative therapy for MHC class II deficiency but it is difficult as affected children have significant comorbidities at the time of HCT. Optimization organ function, implementing a reduced toxicity conditioning regimen, improved T-cell depletion techniques using serotherapy and graft manipulation, vigilant infection surveillance, pre-emptive and aggressive therapy for infection and newer treatments for graft-versus-host disease have improved the transplant survival for children with MHC class II deficiency. Despite persistent low CD4+ T-lymphopenia reported in post-HCT patients, transplanted patients show normalization of antigen-specific T-lymphocyte stimulation and antibody production in response to immunization antigens. There is a need for a multi-center collaborative study to look at transplant survival of HCT and long-term disease outcome in children with MHC class II deficiency in the modern era of HCT.

## Keypoints

- MHC class II deficiency is a form of severe combined immunodeficiency—urgent referral should be made once the diagnosis is suspected.
- Severe and chronic viral infections are the hallmarks of the disease.
- The most prominent immunologic features are the absence or very low HLA-DR expression on lymphocytes, with reduced CD4+ T-lymphocyte counts leading to an inverted CD4/CD8 ratio.
- Early diagnosis of MHC class II deficiency is important to enable prompt referral to a specialized center for hematopoietic cell transplantation before occurrence of end organ damage secondary to recurrent infection.

## Introduction

Major histocompatibility complex (MHC) class II deficiency, also known as bare lymphocyte syndrome type II, is a rare autosomal recessive combined immunodeficiency and was first described in 1980s (1). MHC class II molecules are pivotal for the adaptive immune system and guide the development and function of CD4+ T-lymphocytes. The immunologic hallmark of the disease is the absence of constitutive and inducible expression of MHC class II molecules on all cell types which leads to impaired antigen presentation by HLA-DR, HLA-DQ, and HLA-DP molecules on antigen presenting cells (APC) (2). Besides affecting marrow-derived cells, the lack of MHC class II expression on thymic epithelium also leads to delayed and incomplete maturation of CD4+ T-lymphocytes. MHC class II-mediated peptide presentation is essential for positive and negative selection of the CD4+ T-lymphocyte population in thymus, and for the homeostasis of the mature CD4+ T-lymphocyte population in the periphery. Overall, MHC class II deficiency leads to combined immunodeficiency with defective CD4+ T-lymphocyte maturation and activation and a lack of T helper lymphocyte-dependent antibody production by B-lymphocytes, resulting in significant susceptibility to severe infections and frequently death in early childhood (3). The reported incidence of MHC class II deficiency ranges from 5% of SCID in Canada to 20–30% of SCID in Kuwait and in North Africans Countries (4, 5).

## MHC Class II Deficiency and Genetics

**Figure 1:**
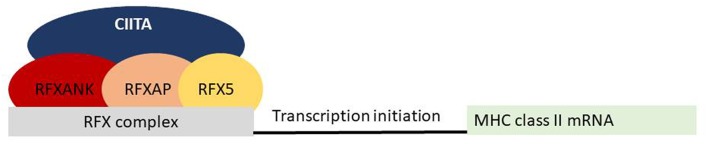
Molecular defects in patients with MHC class II deficiency. Four different transcription factors on MHC class II promoters have been associated with MHC class II deficiency.

The MHC class II genes are located on chromosome 6 and transcription is tightly regulated according to a strict-cell-type-specific and quantitatively modulated pattern. Their expression is largely restricted to thymic epithelial cells and APC that are dendritic cells, macrophages and B-lymphocytes. In MHC class II deficiency, the MHC locus itself is intact in patients and it is a monogenic disease caused by mutations in the genes encoding for four regulatory factors controlling transcription of MHC class II genes. These regulatory factors are CIITA (class II transactivator), RFX5 (regulatory factor 5), RFXAP (RFX-associated protein), and RFXANK (RFX-associated ankyrin-containing protein) (Figure 1).

*CIITA*, which accounts for 15% of MHC class II deficiency, is an inducible factor that controls the expression of MHC class II gene expression by binding to the RFX complex and triggering transcription. The RFXANK, RFX5, and RFXAP proteins are subunits of the ubiquitously expressed RFX complex, which binds directly to the promoters of all MHC class II genes and together with other pleiotropic factors, forms the MHC class II expression enhanceosome (6). Over 85% of affected children have mutations in genes encoding for RFX complex and half of the reported cases have *RFXANK* deficiency.

## Spectrum of Disease in Children With MHC Class II Deficiency

Although MHC class II deficiency is not considered a classical severe combined immunodeficiency (SCID) according to the International Union of Immunological Societies (IUIS) classification criteria, patients usually present with a clinical phenotype that is very similar to SCID (7). Infectious susceptibility is broad toward viruses (e.g., cytomegalovirus, herpes simplex), bacteria (e.g., *Staphylococcus* sp., *Streptococci* sp., *Pseudomonas* sp., *Salmonella* sp.), fungi (e.g., *Candida* sp.), and protozoa (e.g., *Pneumocystis jirovecii)*. These infections affect the gastrointestinal, pulmonary, respiratory tracts, beginning in the first year of life. Severe and chronic viral infections are the hallmarks of the disease and are associated with a poor prognosis. Recurrent bronchopulmonary infections caused by bacteria, viruses and *Pneumocystis jirovecii* are frequently observed. Older children may present with organ impairments such as chronic lung disease, chronic diarrhea with malabsorption and growth faltering. Intestinal and hepatic involvement caused by *Crytosporidium* colonization has been reported in patients with MHC class II deficiency; patients may develop chronic liver disease secondary to *cryptosporidium* infection. The absence of generalized BCGitis in these patients might in part be accounted for by the presence of residual immunity in the form of CD8^+^ T-lymphocytes and natural killer cells. Autoimmune manifestations such as autoimmune cytopenia have been observed in 20% of patients with MHC class II deficiency (8).

## Diagnosis and Immunologic Features of Children With MHC Class II Deficiency

**Figure 2:**
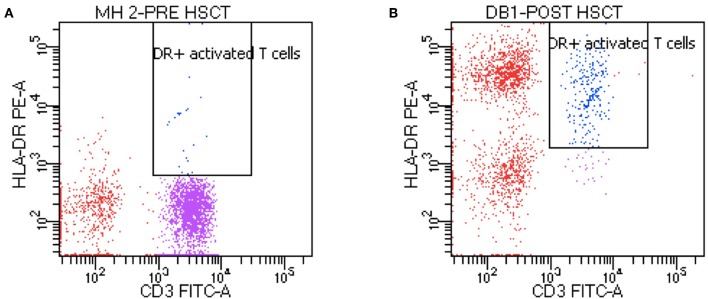
Flow cytometry of a patient MHC class II deficiency. Pre-transplant **(A)** flow cytometry shows absence HLA-DR and post-transplant **(B)** flow cytometry shows presence of HLA-DR.

Patients with MHC class II deficiency generally have severe CD4+ T-lymphocytopenia, hypogammaglobulinemia and lack of antigen-specific antibody responses. Proliferations to mitogen are usually conserved while absent to antigen. The hallmark finding on lymphocyte phenotypes is the absence or very low HLA-DR expression on lymphocytes, with decreased CD4+ T-lymphocyte counts leading to an inverted CD4/CD8 ratio (Figure 2). The CD4+ lymphocytopenia reflects the abnormal CD4+ thymocyte development, resulting from defective MHC class II expression in the thymus. CD8+ T-lymphocyte counts may be normal or low. T cell receptor excision circles (TREC) has been reported to be measurable in some affected patients and the diagnosis can be missed in TREC-based newborn screening for severe combined immunodeficiency (9–11).

## Approach to Haematopoietic Cell Transplantation in Children With MHC Class II Deficiency

The natural history of non-transplanted patients is dismal with a mean age of death at 4 years of age and the main cause of death is overwhelming viral infection (12). Very few children reach puberty and survive into adulthood (13). There are no clear differences in prognosis among patients harboring the four different genetic defects. Currently the only known cure for MHC class II deficiency is allogeneic hematopoietic cell transplantation (HCT). Historically this has only been reluctantly offered due to the high risk of transplant-related morbidity and mortality. Additionally, HCT for MHC class II deficiency is challenging as many children have significant comorbidities at the time of HCT. Transplant strategies to optimize the transplant survival of patients with MHC class II deficiency can be divided into three phrases: (1) pre-transplant phase; (2) transplant phase; and (3) post-transplant phase.

## Pre-Transplant Phase

As younger age at HSCT has been consistently shown to be associated with improved survival in children with primary immunodeficiency, HCT should be performed as early as possible before the onset of organ damage from multiple infections. Some patients with MHC class II deficiency can be detected used TREC-based newborn screening assays, and the diagnosis confirmed by looking for MHC class II expression (9–11). Once the diagnosis of MHC class II deficiency is suspected, a child should be referred promptly to an expert team for evaluation and confirmation of the diagnosis. The transplant process should be initiated and performed as soon as possible. Patients might require treatment of infections, respiratory supports and nutritional rehabilitation to optimize their organ function prior to HCT. A multidisciplinary team with participation of respiratory physicians, gastroenterologists, dietitians, play therapies and other supportive groups are required in all the phases in order to achieve the best outcome possible.

## Transplant Phase

This consists of donor selection, appropriate stem cell source and optimal conditioning regimen. As graft-vs. -host disease confers no benefit to patients with MHC class II deficiency, the best HLA-matched donor is a sibling or matched family donor. If no family donor is found, a search of the national or international unrelated donor registries should be undertaken. Parental haploidentical donors with newer methods of T-lymphocyte depletion have emerged as promising alternative donors while classic haploidentical HSCT with CD34+ selection have shown high rate of non-engraftment in historical series (13–16).

The use of myeloablative reduced-toxicity conditioning (RTC) is preferred in children with MHC class II deficiency as many patients have multiple chronic infections and organ damage at the time of HCT. RTC reduces early transplant complications and late effects such as infertility. In addition, full donor chimerism is not mandatory since stable mixed chimerism on lymphoid and myeloid compartments have been reported to achieve long-term cures. Experiences with reduced intensity conditioning (RIC) are limited. Al-Mousa et al. reported 12 patients transplanted mainly with intra-familial geno-identical donor after a RIC (flu-mel and ATG) and reported mixed lymphoid and myeloid chimerism in all patients but sufficient to cure the disease (17). Outside a geno-identical donor, RIC needs to be used with caution. Based on the current evidence, the European Society for Blood and Marrow Transplant developed a guideline for patients with primary immunodeficiencies (PID) (18). Bone marrow has been the conventional source of stem cells but peripheral blood stem cells have been increasingly used together with RTC to improve donor chimerism (19).

## Post-Transplant Phase

In this phase, the main tasks are monitoring of donor graft, surveillance of transplant-related complications and rehabilitation of pre-transplant organ damage.

## Outcome and Immune Reconstitution After Haematopoietic Cell Transplantation in Children With MHC Class II Deficiency

More than 100 transplants have been reported worldwide (Table 1) (3, 14, 15, 17, 20, 21, 23–26). Patients with MHC class II deficiency are difficult to transplant with increased regimen-related toxicities, serious infections, graft rejection, and GvHD. The conditioning protocols used in these patients were variable and the majority of patients were conditioned with Busulfan-based myeloablative conditioning. In the early reports, the transplant survival was poor compared to those seen in patients with other PIDs, with a survival rate of 50% or less (3, 14, 21, 23, 24). Recently, a better transplant outcome has been reported, the overall survival ranges 66–100% and reduced toxicity conditioning has been increasingly used in these reports. (15, 17, 26). A high incidence of acute graft-versus-host disease has been reported in these patients and this can be accounted by lack of *in-vivo* T-lymphocyte depletion with serotherapy. High rate of chronic persistent viral infections before transplantation may also increase the risk of GVHD. Patients undergoing HCT before 2 years of age had better prognosis (14).

**Table 1:** Outcome of allogeneic hematopoietic cell transplantation for MHC class II deficiency.

**References**	**Year of HCT**	**No. of patients**	**Median age at HCT, months (range)**	**Donor and stem cell source**	**Conditioning regimen**	**GvHD prophylaxis**	**Stem cell doses**	**Grade II-IV aGvHD**	**cGvHD**	**OS (%)**
Elfeky et al. (19)	NA	6	25 (12–38)	2 10/10 UCB 4 9/10 UCB	Flu 150 mg/m^2^ Treo 42 g/m^2^ No serotherapy	CSA+MMF	Median CD34 2.1 × 10^5^/kg	4 (67%)	2	100
Small et al. (15)	1990–2013	16	12 (6–48)	10 MMFD 4 MUD 2 UCB 5 marrow 2 PBSC 1 TCD marrow 6 TCD PBSC	TCD marrow/PBSC Bu 16 mg/kg + Flu + Thiotepa or Bu 16 mg/kg + Thiotepa + Cy Bu 16 mg/kg + Flu + Cy Bu 16 m/kg + CyUnmodified marrow/PBSC Bu + Flu + Cy or Thiotepa +Cy or Melphalan + Flu + anti-CD52 Flu + Cy, anti-CD45 and anti-CD52 or Low dose Cy + Flu + 200cGy TBI UCB Flu + Treo without serotherapy	CSA + MMF	NA	4 (25%)	1 post 2nd HCT	69
Al-Mousa et al. (17)	1994–2007	30 (3 had second HCT)	27 (1–120)	26 MFD marrow 3 MMFD marrow 1 UCB	Bu 16 mg/kg + Cy 200 mg/kg + VP16 300 mg/m^2^ or Bu 16 mg/kg +Cy 200 mg/kg + ATG Cy 200 mg/kg + TBI or Flu 150 mg/m^2^ + melphalan 140 mg/m^2^ +ATG	CSA + MTX or CSA or CSA + steroid	Median CD34 8.3 × 10^6^/kg (3–20.7 × 10^6^/kg)	MAC 9 grade II-III skin aGvHD 5 grade II-III gut aGvHD 1 lung GvHDRIC 7 grade I-II aGvHD	3	66
Siepermann et al. (20)	NA	1	18	7/10 UCB	Bu 20 mg/kg + Flu 160 mg/m^2^ + Cy 120 mg/kg + ATG	CSA	TNC 9 × 10^7^/Kg	Grade I aGvHD	No	Alive
Renella et al. (21)	1981–2004	15 (2 had second HCT)	18 (4–65)	13 MFD marrow 2 MUD marrow All 2 second HCT used MSD	Bu 16–20 mg/kg + Cy 200 mg/kg + ATG in MUD	CSA + MTX	Median TNC: 4.3 × 10^9^/kg (3.3 × 10^8^/kg to 5.5 × 10^9^/kg)	7 (47)	2	53
Saleem et al. (3)	1991–1999	6 (2 had second HCT)	6.5 (1–15.6 years)	5 MFD 1 MUD	Bu 16–20 mg/kg + Cy 200 mg/kg ± Alemtuzumab ± ATG ± anti-LFA-1/CD2 or Flu + Melphalan	CSA	Median TNC: 5.9 × 10^8^/kg (1–11.510^8^/kg)	NA	NA	33
Godthelp et al. (22)	1993–1995	2	8, 23	2 MFD marrow	Bu 20 mg/kg + Cy 200 mg/kg	CSA + MTX	TNC 2.5–4.6 × 10^8^/kg	None	None	Both alive
Bonduel et al. (23)	1994	1 (had 2 HCT)	22	MMUD TCD marrow	Bu 20 mg/kg + Cy 200 mg/kg + anti-LFA1 + anti-CD2	CSA	TNC 0.2 × 10^8^/kg	Grade II GvHD	None	Alive after second sibling CBT
Klein et al. (12)	1981–1993	19 (7 had second HCT)	17 (6–117)	8 MFD marrow 1 MMFD marrow 10 HID marrow All 7 second HCT used HID	MFD Bu20 mg/kg + Cy 200 mg/kg or Cy 50 mg/kg + ALG or Cy 50 mg/kg + CCNU 300 mg/m^2^ + procarbazine 280 mg/kg + ALG MMFD Bu 16 mg/kg + Cy 200 mg/kg or Bu 20 mg/kg + Cy 200 mg/kg + anti-LFA-1 antibody or Bu 20 mg/kg + Cy 200 mg/kg + anti-LFA-1 antibody + anti-CD2 antibody	CSA+MTX	TNC 0.6–6.2 × 10^8^/kg	6 had Grade II-IV GvHD	NA	47
Fischer et al. (24)	NA	20	NA	8 MFD marrow 12 MMFD marrow	Bu 8–20 mg/kg Cy 200 ml/kg ±VP16 900 mg/m^2^	MTX ± CSA	TNC <4 × 10^8^/kg	NA	NA	35

*ALG, antilymphocyte globulin; ATG, Antithymocyte globulin; Bu, Busulfan; Cy, cyclosphosphamide; Flu, Fludarabine; Treo, Treosulfan; VP16, etoposide; CSA, ciclosporin; MMF, mycophenolate mofetil; HCT, hematopoietic cell transplantation; HID, haploidentical donor; MFD, Matched family donor; MMFD, mismatched family donor; MD, matched donor; MMD, mismatched donor; PBSC, peripheral blood stem cell; UCB, unrelated cord blood; TCD, T-cell depletion; aGvHD, acute graft-versus-host disease; cGVHD, chronic graft-versus-host disease; OS, overall survival*.

Among HSCT long-term survivors, persistent CD4+ T-lymphocytopenia has been observed in post-HCT patients. This observation can ben explained by impaired thymic maturation resulting from defective MHC class II expression on thymic epithelia. Although the CD4+ T-lymphocyte number is low, transplanted patients show normalization of antigen-specific T-lymphocyte stimulation and antibody production in response to vaccination. Partial engraftment post-HCT has been associated with impaired immune repertoire (22).

## Summary

MHC class II deficiency is rare but invariably fatal primary immunodeficiency. Allogenic HCT is the only curative treatment and is considered the treatment of choice. Pre-transplant treatment of infections and optimization of nutritional status and organ function are important to improve transplant survival. Transplant survival has improved with reduced toxicity conditioning regimen, better donor availability, improved supportive care, and more effective anti-microbial therapy. There is a need for a multi-center study to delineate the predictors of outcome of HCT in MHC class II deficiency in the modern era of HCT. Advances in gene therapy may be attractive as a potential therapeutic alternative for children with MHC class II deficiency (27, 28).

## Author Contributions

SL performed the literature review and prepared the manuscript. MS, BN, and AG critically reviewed the manuscript.

### Conflict of Interest

The authors declare that the research was conducted in the absence of any commercial or financial relationships that could be construed as a potential conflict of interest.

## References

[1] Lisowska-Grospierre B Durandy A Virelizier JL Fischer A Griscelli C. Combined immunodeficiency with defective expression of HLA: modulation of an abnormal HLA synthesis and functional studies. Birth Defects Orig Artic Ser. (1983) 19:87–91. 6197113

[2] Mach B Steimle V Martinez-Soria E Reith W. Regulation of MHC class II genes: lessons from a disease. Annu Rev Immunol. (1996) 14:301–31. 10.1146/annurev.immunol.14.1.301 8717517

[3] Saleem MA Arkwright PD Davies EG Cant AJ Veys PA. Clinical course of patients with major histocompatibility complex class II deficiency. Arch Dis Child. (2000) 83:356–9. 10.1136/adc.83.4.356 10999878 PMC1718526

[4] Rozmus J Junker A Thibodeau ML Grenier D Turvey SE Yacoub W. Severe combined immunodeficiency (SCID) in Canadian children: a national surveillance study. J Clin Immunol. (2013) 33:1310–6. 10.1007/s10875-013-9952-8 24122030 PMC7102302

[5] Al-Herz W Alsmadi O Melhem M Recher M Frugoni F Notarangelo LD. Major histocompatibility complex class II deficiency in Kuwait: clinical manifestations, immunological findings and molecular profile. J Clin Immunol. (2013) 33:513–9. 10.1007/s10875-012-9831-8 23143406

[6] Reith W LeibundGut-Landmann S Waldburger JM. Regulation of MHC class II gene expression by the class II transactivator. Nat Rev Immunol. (2005) 5:793–806. 10.1038/nri1708 16200082

[7] Picard C Bobby Gaspar H Al-Herz W Bousfiha A Casanova JL Chatila T. International union of immunological societies: 2017 primary immunodeficiency diseases committee report on inborn errors of immunity. J Clin Immunol. (2018) 38:96–128. 10.1007/s10875-017-0464-9 29226302 PMC5742601

[8] Hanna S Etzioni A. MHC class I and II deficiencies. J Allergy Clin Immunol. (2014) 134:269–75. 10.1016/j.jaci.2014.06.001 25001848

[9] Lev A Simon AJ Broides A Levi J Garty BZ Rosenthal E. Thymic function in MHC class II-deficient patients. J Allergy Clin Immunol. (2013) 131:831–9. 10.1016/j.jaci.2012.10.040 23228244

[10] Marcus N Stauber T Lev A Simon AJ Stein J Broides A. MHC II deficient infant identified by newborn screening program for SCID. Immunol Res. (2018) 66:537–42. 10.1007/s12026-018-9019-2 30084052

[11] Aluri J Gupta M Dalvi A Mhatre S Kulkarni M Hule G. Clinical, immunological, and molecular findings in five patients with major histocompatibility complex class II deficiency from India. Front Immunol. (2018) 9:188. 10.3389/fimmu.2018.00188 29527204 PMC5829618

[12] Klein C Lisowska-Grospierre B LeDeist F Fischer A Griscelli C. Major histocompatibility complex class II deficiency: clinical manifestations, immunologic features, and outcome. J Pediatr. (1993) 123:921–8. 10.1016/S0022-3476(05)80388-9 8229525

[13] Ouederni M Vincent QB Frange P Touzot F Scerra S Bejaoui M. Major histocompatibility complex class II expression deficiency caused by a RFXANK founder mutation: a survey of 35 patients. Blood. (2011) 118:5108–18. 10.1182/blood-2011-05-352716 21908431

[14] Klein C Cavazzana-Calvo M Le Deist F Jabado N Benkerrou M Blanche S. Bone marrow transplantation in major histocompatibility complex class II deficiency: a single-center study of 19 patients. Blood. (1995) 85:580–7. 10.1182/blood.V85.2.580.580 7812013

[15] Small TN Qasim W Friedrich W Chiesa R Bleesing JJ Scurlock A. Alternative donor SCT for the treatment of MHC class II deficiency. Bone Marrow Transplant. (2013) 48:226–32. 10.1038/bmt.2012.140 23000650

[16] Shah RM Elfeky R Nademi Z Qasim W Amrolia P Chiesa R. T-cell receptor alphabeta(+) and CD19(+) cell-depleted haploidentical and mismatched hematopoietic stem cell transplantation in primary immune deficiency. J Allergy Clin Immunol. (2018) 141:1417–26.e1. 10.1016/j.jaci.2017.07.008 28780238

[17] Al-Mousa H Al-Shammari Z Al-Ghonaium A Al-Dhekri H Al-Muhsen S Al-Saud B. Allogeneic stem cell transplantation using myeloablative and reduced-intensity conditioning in patients with major histocompatibility complex class II deficiency. Biol Blood Marrow Transplant. (2010) 16:818–23. 10.1016/j.bbmt.2010.01.002 20079864

[18] Effros RB Dillard L Zeller E Naeim F Walford RL. Strong HLA-DR expression in T cell cultures after activation is necessary for IL-2-dependent proliferation. Hum Immunol. (1983) 8:249–54. 10.1016/0198-8859(83)90051-4 6606636

[19] Slatter MA Rao K Abd Hamid IJ Nademi Z Chiesa R Elfeky R. Treosulfan and fludarabine conditioning for hematopoietic stem cell transplantation in children with primary immunodeficiency: UK experience. Biol Blood Marrow Transplant. (2018) 24:529–36. 10.1016/j.bbmt.2017.11.009 29155317

[20] Siepermann M Gudowius S Beltz K Strier U Feyen O Troeger A. MHC class II deficiency cured by unrelated mismatched umbilical cord blood transplantation: case report and review of 68 cases in the literature. Pediatr Transplant. (2011) 15:E80–6. 10.1111/j.1399-3046.2010.01292.x 20214747

[21] Renella R Picard C Neven B Ouachee-Chardin M Casanova JL Le Deist F Human leucocyte antigen-identical hematopoietic stem cell transplantation in major histocompatiblity complex class II immunodeficiency: reduced survival correlates with an increased incidence of acute graft-versus-host disease and pre-existing viral infections. Br J Haematol. (2006) 134:510–6. 10.1111/j.1365-2141.2006.06213.x 16848795

[22] Godthelp BC van Eggermond MC Peijnenburg A Tezcan I van Lierde S van Tol MJ. Incomplete T-cell immune reconstitution in two major histocompatibility complex class II-deficiency/bare lymphocyte syndrome patients after HLA-identical sibling bone marrow transplantation. Blood. (1999) 94:348–58. 10.1182/blood.V94.1.348.413k05_348_358 10381532

[23] Bonduel M Pozo A Zelazko M Raslawski E Delfino S Rossi J. Successful related umbilical cord blood transplantation for graft failure following T cell-depleted non-identical bone marrow transplantation in a child with major histocompatibility complex class II deficiency. Bone Marrow Transplant. (1999) 24:437–40. 10.1038/sj.bmt.1701915 10467337

[24] Fischer A Landais P Friedrich W Gerritsen B Fasth A Porta F. Bone marrow transplantation (BMT) in Europe for primary immunodeficiencies other than severe combined immunodeficiency: a report from the European Group for BMT and the European Group for Immunodeficiency. Blood. (1994) 83:1149–54. 10.1182/blood.V83.4.1149.1149 8111055

[25] Godthelp BC Van Eggermond MC Van Tol MJ Vossen JM van den Elsen PJ. T cell immune reconstitution after allogeneic bone marrow transplantation in bare lymphocyte syndrome. Hum Immunol. (2000) 61:898–907. 10.1016/S0198-8859(00)00156-7 11053633

[26] Elfeky R Furtado-Silva JM Chiesa R Rao K Lucchini G Amrolia P. Umbilical cord blood transplantation without in vivo T-cell depletion for children with MHC class II deficiency. J Allergy Clin Immunol. (2018) 141:2279–82 e2. 10.1016/j.jaci.2017.10.051 29366700

[27] Matheux F Villard J. Cellular and gene therapy for major histocompatibility complex class II deficiency. News Physiol Sci. (2004) 19:154–8. 10.1152/nips.01462.2003 15143213

[28] Booth C Gaspar HB Thrasher AJ. Treating immunodeficiency through HSC gene therapy. Trends Mol Med. (2016) 22:317–27. 10.1016/j.molmed.2016.02.002 26993219

